# Neural network methods for diagnosing patient conditions from cardiopulmonary exercise testing data

**DOI:** 10.1186/s13040-022-00299-6

**Published:** 2022-08-13

**Authors:** Donald E. Brown, Suchetha Sharma, James A. Jablonski, Arthur Weltman

**Affiliations:** 1 School of Data Science, University of Virginia, Charlottesville, VA USA; 2 Department of Engineering Systems and Environment, University of Virginia, Charlottesville, VA USA; 3 Department of Kinesiology, University of Virginia, Charlottesville, VA USA; 4 Division of Endocrinology and Metabolism, Department of Medicine, University of Virginia, Charlottesville, VA USA

**Keywords:** Machine learning, Convolutional neural networks, AutoencoderClassifier

## Abstract

**Background:**

Cardiopulmonary exercise testing (CPET) provides a reliable and reproducible approach to measuring fitness in patients and diagnosing their health problems. However, the data from CPET consist of multiple time series that require training to interpret. Part of this training teaches the use of flow charts or nested decision trees to interpret the CPET results. This paper investigates the use of two machine learning techniques using neural networks to predict patient health conditions with CPET data in contrast to flow charts. The data for this investigation comes from a small sample of patients with known health problems and who had CPET results. The small size of the sample data also allows us to investigate the use and performance of deep learning neural networks on health care problems with limited amounts of labeled training and testing data.

**Methods:**

This paper compares the current standard for interpreting and classifying CPET data, flowcharts, to neural network techniques, autoencoders and convolutional neural networks (CNN). The study also investigated the performance of principal component analysis (PCA) with logistic regression to provide an additional baseline of comparison to the neural network techniques.

**Results:**

The patients in the sample had two primary diagnoses: heart failure and metabolic syndrome. All model-based testing was done with 5-fold cross-validation and metrics of precision, recall, F1 score, and accuracy. As a baseline for comparison to our models, the highest performing flow chart method achieved an accuracy of 77%. Both PCA regression and CNN achieved an average accuracy of 90% and outperformed the flow chart methods on all metrics. The autoencoder with logistic regression performed the best on each of the metrics and had an average accuracy of 94%.

**Conclusions:**

This study suggests that machine learning and neural network techniques, in particular, can provide higher levels of accuracy with CPET data than traditional flowchart methods. Further, the CNN performed well with a small data set showing that these techniques can be designed to perform well on small data problems that are often found in health care and the life sciences. Further testing with larger data sets is needed to continue evaluating the use of machine learning to interpret CPET data.

## Background

Research over the last four decades has given us conclusive evidence that physical activity plays a major role in the prevention and treatment of chronic diseases, with at least a quarter of million deaths per year in the U.S. in 2003 attributable to a lack of cardiorespiratory fitness [1–3]. Cardiopulmonary exercise testing (CPET) provides an objective, reliable, and reproducible assessment of cardiorespiratory fitness and, as such, represents an effective instrument for use by clinical practitioners to improve the health outcomes of their patients [4, 5]. CPET measures physiological response to physical exercise through an array of pulmonary, cardiovascular and metabolic measurements, built around breath-by-breath gas exchange analysis. These measurements produce multivariate time-series that when viewed by trained personnel can yield understanding of health and disease through the interpretation of complex and dynamic ventilatory, cardiovascular and gas exchange variables across a dynamic range of external power outputs.

Unfortunately, the amount and form of data provided by CPET means that it is difficult to understand and, hence, use in clinical practice [6–8]. To address this problem researchers developed flowcharts, which have become a well-documented and commonly taught approach to using CPET to determine the pathophysiology of a patient [9]. Flowcharts allow the health care practitioner to follow a series of binary, branching questions to produce an interpretation or diagnosis. However, flowcharts have been around for more than 20 years [10] and CPET remains underutilized and difficult to interpret.

This paper provides a first study to determine if machine learning methods improve upon flowcharts as way to obtain diagnostic information from CPET. Specifically, the paper compares deep learning neural networks and a simpler regression-based approach to flowcharts for integrating CPET results from a small number of cases to produce diagnostic assessments. The small size of the data set is important for this study because it often assumed that deep learning approaches are not suitable for health care problems, like this one, with small numbers of well-labeled data. Hence, this research is also important for assessing the feasibility of using these new methods in health care.

**Fig. 1:**
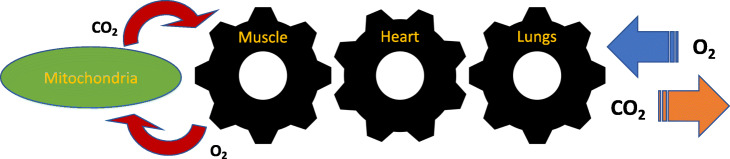
Coupling of external to cellular respiration. Adapted from Wasserman K. Am J Physiol.1994 Apr;266(4 Pt 1):E519–39.(2)

Interpretation of CPET data derives from the biochemical processes that transform energy into physical movement. These processes efficiently link the O_2_ and CO_2_ gas transport among three interconnected organ systems at the core of exercise responsiveness and capacity: pulmonary, cardiac, and skeletal muscle [11, 12] (Fig. 1). The pulmonary system (lungs) transfers inspired O_2_ from the air to deoxygenated blood in pulmonary circulation. The cardiac system (heart) then pumps oxygenated blood from pulmonary circulation to peripheral circulation.

Skeletal muscle mitochondria utilize O_2_ derived from the peripheral circulation for aerobic cellular energy metabolism to fuel movement and locomotion. CO_2_ generated from cellular metabolism is then transferred in reverse order from skeletal muscle back to the heart and is subsequently expired from the lungs. Given the interconnectedness from internal to external respiration, CPET assessment of the rate of inspired volumes of oxygen ($\dot {V}\mathrm {O}_{2}$ mL/min) and expired carbon dioxide ($\dot {V}\text {CO}_{2}$ mL/min) can identify small perturbations in each of these vital organ systems. CPET analysis can thus yield insight into the underlying causes for clinical manifestations of dyspnea, fatigue, and associated exercise intolerance.

CPET clinical assessment of simultaneously generated heart rate (HR beats/min), ventilation, and gas exchange data is reported as averaged values over fixed intervals (e.g., every 30 seconds for the duration of a 12 ±6 minute test). Tabular CPET data are used to identify key physiologic responses to exercise at test end. For example, CPET measurement of the highest rate of oxygen consumption during progressive exercise normalized to body mass (peak $\dot {V}\mathrm {O}_{2}$ mL/min ·kg) is the gold standard assessment of cardiorespiratory fitness. Importantly, peak $\dot {V}\mathrm {O}_{2}$ is one of the strongest predictors of health outcomes and mortality across various patient populations [13–15]). Peak $\dot {V}\mathrm {O}_{2}$ is also used in specific clinical scenarios such as the evaluation of response to therapies in heart failure patients being considered for transplant [16–18]. In addition to CPET data collected at peak exercise, submaximal data collected over the course of CPET provide clinicians with important information regarding a patient’s etiology of exercise limitation [13, 19]. An example is the relationship between pulmonary ventilation (VE mL/min) and $\dot {V}\mathrm {O}_{2}$, where the slope of these parameters (VE/$\dot {V}\text {CO}_{2}$) has strong associations and prognostic implications in systolic heart failure and pulmonary arterial hypertension [3, 9]. The challenge of CPET clinical assessment is that test interpretation requires careful attention to multiple variable relationships simultaneously over time.

**Fig. 2:**
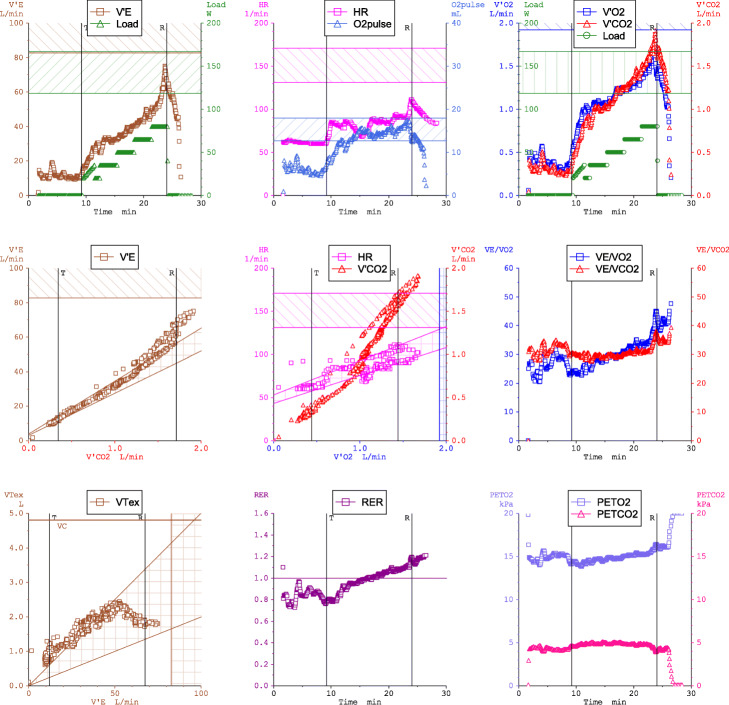
Nine-panel plot cardiopulmonary exercise testing data visualization

To assist analyses of the high volume of multivariate data generated from each test, graphical visualization of CPET data is essential for proper interpretation. For more than 20 years, the most commonly used display for CPET data visualization has been the Wasserman nine-panel plot, which shows multiple channels of data on a one-page summary consisting of 9 distinct scatter plots (Fig. 2) [9, 20]. Using the Wasserman nine-panel plot, flowcharts are used to guide diagnostic and prognostic considerations [9]. Nonetheless, there remains significant inter-individual variability in CPET interpretation [7, 21]. Attempts to refine CPET assessment over the past 30 years have thus far failed to substantially improve the diagnostic power of CPET or simplify interpretation to guide clinical care and promote wider adoption of exercise testing.

Modern machine learning techniques have promise for making CPET more readily accessible to physicians. Akay, et al. [22] developed a support vector regression to predict one of the most used measures of fitness from CPET, the maximum VO_2_ for the person under test. Akay and his colleagues later showed that this task could be done with improved accuracy using neural networks [23]. Neural networks have also been shown to predict coronary artery disease using data from exercise stress testing (a more data intensive test than CPET) [24]. Sakr, et al. [25] compared a number of machine learning techniques with exercise data and showed that machine learning can effectively predict all-cause mortality. A subsequent paper by this group showed that it might be possible to improve the interpretability of machine learning techniques used on exercise data by clinicians [26].

A survey by Javan et al. [27] discusses machine learning applications to cardiac arrest prediction and recommends improvements to external validation of the models. Other researchers [28] hypothesized that heart failure prediction could be improved by considering the totality of the time-series data from CPET, as opposed to summary indices alone. Their best predictive performance was obtained with a neural network that they claimed could be improved with more training data. More recently Shandi, et al. studied the use of a small wearable patch to be worn during CPET that could accurately estimate oxygen uptake [29]. They used a neural network model to classify heart failure risk levels according to the features of gas exchange variables derived from the patch device. Finally, a recent study by Inbar, et al, used support vector machines to obtain good probability estimates for interpretation of CPET results to distinguish among patients with chronic heart failure (CHF) and chronic obstructive pulmonary disease (COPD) [30].

All of this previous research provided motivation for the study described in this paper. We wanted to further determine the usefulness of machine learning techniques to support diagnosis of important medical conditions with a bench mark comparison of the flow sheet methods used by practitioners. We specifically wanted to determine if machine learning, and particularly more modern deep learning approaches, could effectively integrate the multiple streams of CPET results into a coherent estimate of the patient’s condition.

A potential concern for using machine learning, and particularly deep learning, is that many health care problems have small numbers of curated, labeled examples in data sets for specific diseases. This is a notable concern for CPET, where the numbers of cases with curated labels available at many health institutions is small, while the number of potential labels is large. As noted by many researchers in deep learning, these methods are particularly data-hungry requiring thousands, if not millions of labeled training examples [31, 32]. Hence, another motivation for this study was the need to understand if deep learning methods have value for health care problems, such as CPET, where the data have variety in terms of numbers and types of variables, but have limited labeled examples for training and testing.

## Methods

As noted above, the two aims of this study are 1. Use a representative set of CPET results to compare the diagnostic performance of machine learning methods, particularly deep learning, to the standard flowcharts; and 2. Assess the diagnostic performance of deep learning approaches with a small CPET data set. The subsections below describe the data used in the study and the different approaches the study compared for interpreting the results from CPET.

### Data

The data set used in the study consists of anonymized results from CPET of patients with two clinically diagnosed conditions: heart failure and metabolic syndrome^1^.

We obtained the metabolic syndrome data from a study supported by a National Institute of Health/National Heart Lung and Blood Institute (NIH/NHLBI), “Exercise dose and metformin for vascular health in adults with metabolic syndrome." Several papers provide details on this study and the data the researchers obtained [33–35].

The heart failure data came from patient studies supported by the American Heart Association, “Personalized Approach to Cardiac Resynchronization Therapy Using High Dimensional Immunophenotyping,” as well as the NIH/NHLBI, “MRI of Mechanical Activation and Scar for Optimal Cardiac Resynchronization Therapy Implementation.” The primary researchers for these studies have reported their results and described their data in the literature [36–38].

The protocol for the testing in each of these studies used a treadmill with three phases: rest, test, and recovery. During test phase the slope and speed of the treadmill were incrementally increased. The CPET of the patients was performed by the Exercise Physiology Laboratory (EPL) of the General Clinical Research Center (GCRC) at the University of Virginia. This data set contains the CPET results and demographic information for 30 patients with either of the two conditions and there 15 patients with each condition. The anonymized demographic information includes gender, age, height, weight, and body mass index (BMI). The CPET variables collected per patient are shown in Table 1. Since the purpose of our study was to compare machine learning results with those obtained from flowchart analysis, only the data for the CPET variables and not the demographic variables were used as inputs to the approaches described in the next section.

**Table 1:** Features of the CPET data

Feature name	Feature description
Time (min)	Breath-by-Breath.
METS	Metabolic equivalents.
HR	Heart Rate.
$\dot {V}$O_2_(L/min)	Peak oxygen consumption.
$\dot {V}$O_2_/kg((ml/min)/kg)	Peak oxygen consumption is measured in milliliters of oxygen used in one minute per kilogram of body weight.
$\dot {V}$CO_2_(L/min)	Volume of carbon dioxide released.
RER	Respiratory Exchange Ratio.
VE(L/min)	Ventilation.
VE/$\dot {V}$O_2_	Ratio of Ventilation by peak oxygen.
VE/$\dot {V}$CO_2_	Ratio of Ventilation by volume of carbon dioxide released.
RR(L/min)	Respiratory rate.
VTex(L)	Expiratory tidal volume(Expiratory time).
VTin (L)	Inspiratory tidal volume(Inhale time).
Speed(mph)	Speed of the treadmill.
Elevation	Elevation of the treadmill.

### Approaches

This study compares the baseline flowchart approach with four other approaches, one statistical and the other three using neural networks. The statistical approach uses logistic regression with inputs taken from a principal component analysis (PCA) of the CPET results. The neural network methods are autoencoder projection and a convolutional neural networks (CNN). Descriptions of these approaches are below.

#### Flowcharts

**Fig. 3:**
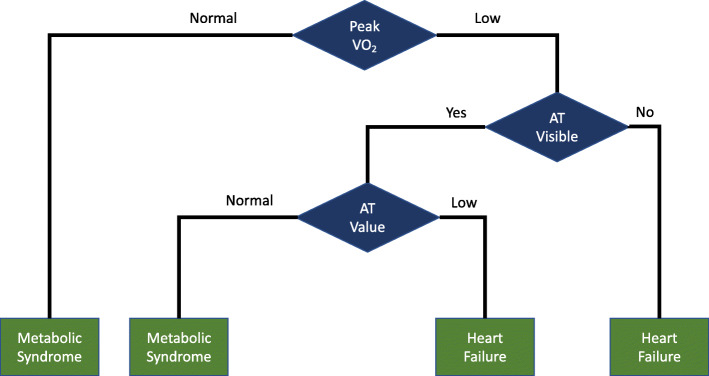
Flowchart for baseline model

The baseline method that is used to interpret CPET results is a flowchart. Flowcharts have been used to interpret CPET results for more than 30 years. The flowchart used for this analysis is derived from the accepted textbooks on CPET [9, 11] and is shown in Fig. 3. This flowchart has been adapted to classify the results from a patient’s CPET into one of the two categories, heart failure or metabolic syndrome. The resulting flowchart shown in Fig. 3 uses both peak $\dot {V}\mathrm {O}_{2}$ and AT measurements to perform this classification. However, to implement the flowchart requires specification of normal values. This study used two commonly accepted sources to specify the normal values for ventilatory thresholds: the Fitness Registry and the Importance of Exercise National Database (FRIEND) [39] and the guidelines from Hansen, et al. [40]. FRIEND was created to provide measures on CPET variables for apparently healthy individuals in the USA. The guidelines in [40] provide specifications for normal patient values for CPET variables. These two different sources for normal values produce two different results for the same flowchart (Fig. 3).

#### PCA-regression

While flowcharts provide a baseline method widely used in practice, logistic regression provides another standard approach that can be applied to CPET results. This study developed a regression model using the variables METS, HR, $\dot {V}\mathrm {O}_{2}, \dot {V}\text {CO}_{2}$, RER, VE, VTex, and VTin, (see Table 1). The response variable was the patient diagnosis: heart failure or metabolic syndrome. To account for the correlation between the CPET variables this research study used principal component analysis (PCA) to project the data onto dimensions (features) that are orthogonal and linearly independent.

The goal of PCA is to find a projection of the data from the original variable space (given by variables in Table 1 into a new, smaller dimensional space composed of axes that are linear combinations of the original features. The goal is to find a projection that minimizes the variance of the data in the projection. So, if $\hat {\Sigma }$ is the sample covariance matrix for the features of the CPET time series, then PCA finds projections that are orthonormal (orthogonal and with unit length). This means maximizing $u_{1}^{T}\hat {\Sigma }u_{1}$, where *u*_1_ is the first principal component. For all other principal components, *i*,*j*, we add the additional constraint that $u_{i}^{T}u_{j} = 0$. For the method used in this study, we projected the data only onto the first three principal components and the used that projection as input to a logistic regression.

From a physiologic standpoint the PCA projection does more than produce orthonormal predictor variables. It also captures the change in correlation among CPET variables as the test proceeds. Essentially the major physiological subsystems of the human body must work together as the well-oiled machine described by Wasserman[11]. If there is impairment in one or more parts of these subsystems, the patient will not cope with the increasing work in the exercise test protocol. The subsystems display differential patterns of volatility that can be detected when projected onto a hyperplane by PCA. The weights or loadings for each of the CPET variables in the PCA projections will change as the correlations between those variables change during each of the stages of the test. Ideally the changes in these PCA projections will capture the clinically relevant features. So for this study to capture the volatility in patient performance to exercise testing, the predictor variables used for the logistic regression are the interquartile ranges for each of the stages of the test as measured in the first three principal components. In other words, we start by finding the first three principal components for each stage of the test and then we obtain the interquartile range (IQR) for these components. These features should effectively capture the relevant differences in the rest, test, and recovery stages for each patient as measured across all of the CPET variables but projected into the first principal component. A single logistic regression then converts the values in this projection into the probability that the correct interpretation of the CPET results is metabolic syndrome or heart failure for each of the patients.

#### Autoencoder-regression

The next approach to CPET interpretation directly extends the PCA-regression approach of the previous subsection by the replacing PCA linear projection of the data with a possibly nonlinear projection produced by an autoencoder. Autoencoders are neural networks with two major components. In the first part, the encoder, maps the data from the CPET variables, METS, HR, $\dot {V}\mathrm {O}_{2}, \dot {V}\text {CO}_{2}$, RER, VE, VTex, and VTin, (see Table 1), using a restricted function that reduces the dimensionality of the data. In this study we used grid search to determine the choices for tuning parameters including the number of principal components and the dimensionality of the middle layer in the autoencoder and observed the best results at three dimensions in both cases. The output from this part is then input to the second part, the decoder, that attempts to reproduce the original data.

Let *x* be the input data, *f* be the encoding function that reduces the dimensionality of the input, and *g* be the decoding function. Then autoencoder learns the weights in the neural network by minimizing the loss function. For this study we used mean squared error,

$$ \frac{\sum_{i=1}^{N} (x_{i} - g(f(x_{i})))^{2}}{N} $$

where *N* is the number of breath-to-breath observations for a patient.

The restriction on the encoder means that the encoding function must learn only the essential features of the data useful in its reproduction. Like PCA the encoder is projecting the data into a reduced space, but unlike PCA the projection can be nonlinear. Autoencoders have a rich history of development and use in the machine learning community [41, 42], as well as, applications for problems in health care, such as, histopathology [43, 44].

**Fig. 4:**
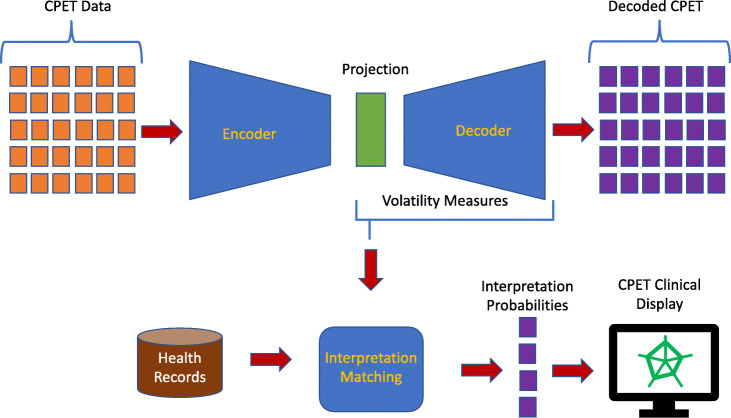
CPET Autoencoder Architecture

Figure 4 shows the architecture developed in this study to transform the CPET data into clinically useful interpretations. In this approach the data provided by CPET are input to an autoencoder neural network. As noted above this component performs the nonlinear projection of the CPET data into the manifold that best encodes the CPET data for accurate decoding. As with PCA the autoencoder projection represents the combined functioning of the physiological subsystems before, during, and after exercising. The projection from the manifold is then aggregated using the same measures of volatility (i.e., interquartile range) that capture and describe the functioning of the physiological systems. As with PCA, this projection reduces the dimensionality of the data into the much smaller space that captures the latent features from the exercise testing. These features again become inputs to a logistic regression which outputs the likelihood of a one of the clinical interpretations.

#### Convolutional neural networks (CNN)

In addition to autoencoders, this study also investigated the use of convolutional neural networks (CNN) for condition diagnosis using CPET data. As the name implies CNN contain specialized mathematical structures (convolutions), designed to learn spatial hierarchies of features [45]. Convolutions consist of three parts: the input, the kernel or the function that operates over the data, and the output or the feature map. In most implementations the kernel function operates over a small region input values. To capture all the input the convolution is applied iteratively across all regions of the input space. Convolutions are efficient and effective methods that allow us to learn structures or identify patterns despite noise and natural variations in the data. CPET output measurements contain many sources of natural variation, such as, measurement noise, changing experimental conditions, and highly varying patient ability levels and physiology. As such, CNN can prove useful at identifying underlying clinically relevant patterns in the CPET time series that may assist with disease diagnosis and understanding.

CNN architectures commonly consist of multiple convolutional layers, as well as, pooling layers that choose a summary value (most often the maximum or the mean) from among the input values for each input region. Through training these layers of a CNN architecture gradually detect more and more complex patterns [46].

Indeed, CNNs have achieved impressive results in variety of health care applications [47–49]. For both univariate and multivariate time-series they demonstrate state-of-the-art performance in both health care and other domains [50]. The highest performing neural net based classifiers are all deep CNNs, and they often require less training and prediction time than other leading time-series classification methods [51]. Sliding windows used in their structures prove effective at recognizing spatial patterns extant in time-series, and the hierarchical nature of deep architectures enables them to learn patterns at varying scales.

Most well-known CNN architectures have been built for image recognition. For example, AlexNet [52], VGG-16 [53] and ResNet-34 [54], each showed excellent performance, in some cases superior to human performance, for detecting patterns in images. The use of CNN architectures for diagnosis and classification from multivariate time series data has a similar set of commonly used architectures. The use of one-sided convolution kernels has been used with health record data to predict the risk of congestive heart failure and chronic obstructive pulmonary disease in patients [55]. The one-sided kernels have also been used to find patterns in multidimensional time series electroencephalographic (EEG) recordings [56, 57]. More recently researchers have used two sided kernels with health record data to predict patient health care costs [58].

**Fig. 5:**
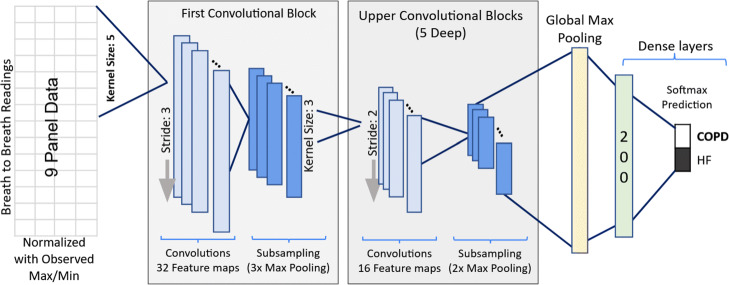
CPET CNN Architecture

This study uses two sided kernels with 8 channels for each of the CPET variables METS, HR, $\dot {V}\mathrm {O}_{2}, \dot {V}\text {CO}_{2}$, RER, VE, VTex, and VTin. Each of theses individual CPET time series are normalized and then presented to the neural network across the 8 channels to capture the multi-dimensional time series output from CPET. Figure 5 shows the CNN architecture we constructed for this. In this architecture the network consists of six layers of one-dimensional convolutional blocks organized from the first to the upper layers of convolutions. Each of the one-dimensional layers allows for movement along the CPET time series with a fixed kernel size (window) and also stride length which determines the amount of overlap in adjoining windows. These convolutional blocks are followed by a global max pooling layer and then two fully connected layers. The pooling layer changes the output at a specified location in the network to the maximum value among all neighbors to that location. The fully connected final layer consists of a softmax function that yields the clinical interpretation and associated probability as was produced by the logistic regression based approaches described in the previous subsections.

For this study we conducted a random search with a single fold of validation data to derive and then confirm the overall network and associated training hyperparameters. For this search we used the Adam optimization algorithm with a learning rate of.0001, a batch size of 4, and early stopping with a patience of 15 epochs and a threshold of.01 for validation loss. We did not employ dropout or batch normalization in any of the layers during training. The kernel lengths at each level are shown in Fig. 5.

## Results

Each of the approaches described in “Approaches” section was evaluated using four metrics: precision, recall, F1 score, and accuracy. Precision is the number of patients for whom a medical condition is correctly classified divided by the number of patients given that classification. Recall is the number of times a medical condition is correctly classified divided by the number of times the condition occurs in the test set. The F1 score combines precision and recall into one score using the harmonic mean of their values. So,

$$ F1 = 2 \cdot \frac{\text{Precision}\cdot\text{Recall}}{\text{Precision}+\text{Recall}} $$

Finally, accuracy is simply the number of times that the patients were correctly classified divided by the number of patients in the set.

Because of the small number of patient CPET results with clinically labeled classifications, this study used 5-fold cross validation to assess the performance of each of the approaches. 5-Fold cross validation means that we randomly divided the dataset into 5 parts. We then trained each method on 4/5 of the dataset, tested it on the remaining 1/5 of the dataset, and repeated this 5 times. The results are the average performance of the 5 tests.

Table 2 shows the 5-fold cross-validation results for each of the approaches. The flowchart methods have very good precision values on differing conditions, but their accuracy for these data is less than 80%. Each of the machine learning approaches outperforms the flowcharts for F1 score and accuracy. The three machine approaches (PCA + logistic regression, Autoencoder + logistic regression and, and CNN) also do well on most of the values of precision and recall. Overall the autoencoder with logistic regression does the best on these data with an accuracy 97% and an F1 score of 0.97. In contrast the both the PCA with regression and the CNN have accuracy values of 0.90 and F1 scores at or below 0.92.

**Table 2:** Results Comparison Table

Model	Condition	Precision	Recall	F1 Score	Accuracy
flowchart (Hansen)	Heart Failure	1.00	0.53	0.70	70
	MetSyn	0.76	0.87	0.81	
flowchart (FRIEND)	Heart Failure	0.78	0.93	0.85	77
	MetSyn	1.00	0.60	0.75	
PCA + Logistic Regression	Heart Failure	0.93	0.87	0.90	90
	MetSyn	0.88	0.93	0.90	
AE +Logistic Regression	Heart Failure	0.94	1.00	0.97	97
	MetSyn	1.00	0.93	0.97	
CNN	Heart Failure	1.00	0.80	0.86	90
	MetSyn	1.00	1.00	0.92	

## Discussion

Cardiopulmonary Exercise Testing (CPET) is considered the most effective and available current technique to measure excise fitness in patients as evidence for diagnosis [4, 5]. This study investigated the use of neural networks approaches to improve upon the standard flowchart method for diagnosing patient conditions using results from CPET. Flowcharts have been used to interpret CPET results almost since the first medical uses of CPET and remain a basic element of CPET interpretation training programs for physicians in clinical practice, academics, exercise scientists, and laboratory personnel [9]. This question posed by this study is: Can modern machine learning methods provide more accurate classifications of underlying patient conditions using only the CPET data than the classifications provided by the flowchart method?

To conduct this investigation this study used data from 30 patients who underwent CPET testing at the Exercise Physiology Laboratory (EPL) of the General Clinical Research Center (GCRC) at the University of Virginia. The patients were evenly split in the diagnoses between heart failure and metabolic syndrome. The small size of the data set is important for this study because it often assumed that machine learning methods, particularly deep learning like CNN, require large numbers of well-labeled data. Hence, this research is also important for assessing the feasibility of using these new methods in health care.

The study compared two well-known and widely used neural network approaches, autoencoders and convolutional neural networks (CNN), with both the flowchart method and a principal component analysis (PCA) based regression. The principal component regression provides an additional non-neural network as a baseline for the results. Autoencoders are neural networks used for data compression and this makes them ideal for finding latent data structures for diagnosis that might exist within the CPET data. CNN have demonstrated excellent performance with different data types to include images and time series, although typically with considerably more training data than what was available for this study.

The study evaluated each of the methods using 5-fold for cross-validation and the metrics, precision, recall, F1 score, and accuracy. The use of cross-validation provides an out-of-sample test with a small data set. The results showed that the architecture using an autoencoder with logistic regression had the best performance across all metrics. The CNN and the PCA regression produced similar and much better predictions than the flowchart methods.

The performance of the autoencoder versus PCA logistic regression indicates that the CPET data show patterns that are better detected with the non-linear projection of the autoencoder rather than the linear projection of PCA. Intuitively this implies that relationships between the cardio and pulmonary variables recorded during exercise, rest, and recovery vary in nonlinear relationships to each other.

While the performance of the CNN is not as good as that of the autoencoder, it shows remarkably strong performance on a small data set. As one of the objectives of this study was to determine the viability of applying deep learning techniques to typical health studies when there is a small number of labeled results, these results are promising.

There are a number of limitations to this study that require further investigation. This is clearly a very small data set and the results provided can only be considered hypothesis generating. The differences in the CPET data between heart failure and metabolic syndrome patients may be easily detectable by the machine learning methods because the testing protocols used by the lab technicians for these patients was different and not because their underlying conditions were otherwise visible in the data. Only further testing with data from patients with other diseases and health conditions will allow us to explore this. Additionally we did not use synthetic data to increase the number of cases for training. We did this in order to be fair to the flowchart methods, since the synthetic data will enable better training of the machine learning methods, while the flowchart methods will be unaffected. Finally, the choice the neural network techniques and architectures was based on past performance of these methods on similar problems. Further investigation is needed to determine if other neural network approaches could produce even better performance on CPET data.

## Conclusions

This paper investigated whether neural network approaches could improve upon the use of flowcharts with CPET data for the prediction of patient conditions. The results of small sample testing showed that two commonly used neural network approaches, autoencoders and convolutional neural networks, do provide much improved predictions versus the flowchart method. Additionally, the performance of the convolutional neural network was very good despite the small size of the training data set. This suggests that the use of deep learning methods for health care and life science problems may not be handicapped by data set size. Synthetic data for training was not used in this study to allow a fair comparisons of the machine learning techniques with the flowchart methods. Future work could explore the use of synthetic data for training of the neural network. Finally, the overall good performance of the neural networks in this study for predicting patient conditions signals the need for continued testing of these methods with larger and more diverse data sets.

## Data Availability

https://github.com/suchethassharma/CPET.

## Abbreviations

CPET: Cardiopulmonary Exercise Testing
PCA: Principal Component Analysis
CNN: Convolutional Neural Network
EPL: Exercise Physiology Laboratory
GCRC: General Clinical Research Center
UVA: University of Virginia
METS: Metabolic equivalents
HR: Heart Rate
$\dot {V}$O_2_(L/min): Peak oxygen consumption
$\dot {V}$O_2_/kg((ml/min)/kg): Peak oxygen consumption is measured in milliliters of : oxygen used in one minute per kilogram of body weight
$\dot {V}$CO_2_(L/min): Volume of carbon dioxide released
RER: Respiratory Exchange Ratio
VE(L/min): Ventilation
VE/$\dot {V}$O_2_: Ratio of Ventilation by peak oxygen
VE/$\dot {V}$CO_2_: Ratio of Ventilation by volume of carbon dioxide released
RR(L/min): Respiratory rate
VTex(L): Expiratory tidal volume(Expiratory time)
VTin (L): Inspiratory tidal volume(Inhale time)

## Footnotes

1 https://github.com/suchethassharma/CPET
